# Prevention of depression and anxiety symptoms in adolescents: 42 and 54 months follow-up of the Aussie Optimism Program-Positive Thinking Skills

**DOI:** 10.3389/fpsyg.2014.00364

**Published:** 2014-05-28

**Authors:** Julie Johnstone, Rosanna M. Rooney, Shari Hassan, Robert T. Kane

**Affiliations:** Curtin UniversityPerth, WA, Australia

**Keywords:** Aussie Optimism, follow-up, anxiety, depression, child, school-based prevention program, early intervention

## Abstract

Anxiety and depression are the most commonly reported mental health problems amongst Australian children and adolescents. The Aussie Optimism: Program-Positive Thinking Skills (AOP-PTS) is a universal intervention program based on cognitive and behavioral strategies and aimed to prevent anxiety and depression in the middle primary school children aged 9–10 years old. 370 students randomly assigned to the intervention and control condition participated in the 42 and 54 months follow-up study. The intervention group received the AOP-PTS 10-week program and the control group received the regular health education curriculum. Students were assessed on anxiety, depression and attribution style at school whilst parents reported on their child’s externalizing and internalizing problems at home. Results showed there were no significant reductions across groups in the depressive and anxiety symptoms, and attribution style at either 42 or 54 months follow-up. These findings suggest that AOP-PTS has short and medium term effects but were not sustained in longer term period. Future strategies to achieve the desirable outcomes in a longitudinal study are discussed.

## INTRODUCTION

A recent Australian government report cited mental health problems as accounting for the highest burden of disease among Australian children (Bayer et al., 2007; Australian Institute of Health and Welfare, 2009). Whilst externalizing disorders, such as ADHD and conduct disorder, represent the more overt manifestations of mental health problems in children, the internalizing disorders, such as anxiety and depression, are consistently reported as the most common mental health problems amongst Australian children aged between 7 and 14 years (Prior et al., 1999; Australian Institute of Health and Welfare, 2009). Given that internalizing disorders are less easily recognized, it is alarming to note that depression and anxiety disorders amongst children under age 14 represented approximately 13% of presentations to general medical practices in Australia between April 2007 and March 2008 (Australian Institute of Health and Welfare, 2009).

More recently, a Western Australian government report revealed that whilst approximately 30% of children aged up to 15 years were reported by parents as having emotional problems, only an estimated 4% were reported as having received treatment during the past 12 months; the lowest reported rate of treatment interventions since 2002 (Patterson et al., 2012). Evidence consistently indicates that anxiety or depressive symptoms during childhood may develop into similar disorders during adolescence and adulthood (Barrett et al., 1996, 2005; Kovacs, 1997; Bandura et al., 1999; Bayer and Sanson, 2003; Klein et al., 2005) and it was predicted that life time cases of mental health disorders partially started by the age of 14 (Robinson et al., 2011). Further, children who experience anxiety or depression are at increased risk of co-morbidity for other mental health disorders, including conduct disorder and substance use problems (Roberts, 1999). If not prevented, childhood internalizing disorders can become associated with psychosocial problems such as increased alcohol and substance use, higher rates of cigarette smoking and, for females, higher rates of unprotected sex (Robinson et al., 2011). Clearly, the social and economic costs of childhood internalizing disorders extends beyond those associated with a single episode of any disorder. Fortunately recent evidence suggests that an episodic or chronic course of mental health problems may be prevented if internalizing disorders are managed or prevented at an early age (Robinson et al., 2011).

To develop preventive programs it is useful to understand the factors that place children at increased risk of developing internalizing disorders. For example, relationships have been reported between childhood internalizing disorders and low levels of parental acceptance and child self-esteem (Papandrea et al., 2010); and socio-economic variables including low income, single parents, parent education, and parent unemployment (Davis et al., 2010). Davis et al. (2010) proposed that parental unemployment may result in parents adopting a more negative worldview. The parental modeling of negative behaviors in response to life challenges may inadvertently teach children that they are incompetent to deal with difficulties that arise, leaving the children in a state of learned helplessness (Bayer et al., 2010). Similarly, Davis et al. (2010) described a strong relationship between low income and education, and fewer positive parenting behaviors.

A number of factors have been identified to account for the increased psychological stressors arising during adolescence. Kuperminc et al. (2001), for example, commented on the transition away from reliance on parental and adult support to the importance of peer supports during the period of transition to early adolescence (and thus transition to high school). The importance placed on peer relationships, contributes to the increased risk of mental health problems, especially if such relationships are not well developed (Kuperminc et al., 2001).

Eccles et al. (1993) added that the transition to high school can exacerbate psychological vulnerabilities due to the increased focus on performance comparisons amongst students – both academically and socially – at a developmental period when adolescents are very self-conscious. Students who have more positive attributional styles and appropriately directed locus of control may be more resilient to the potentially harmful psychological sequelae during these periods of increased social and academic comparison.

In addition to the afore-mentioned factors associated with parenting styles, modeled behaviors, socio-economic variables, cognitive styles may contribute to development and maintenance of mental health problems. Alloy et al. (1999), for example, reported an association between negative cognitive styles and risk of depression. Others have reported irrational beliefs, negative automatic thoughts, and pessimistic attributions as important risk factors for mental health disorders (Roberts, 1999; Bayer and Sanson, 2003; Roberts et al., 2008). Negative cognitions including poor self-concept, low self-esteem, and negative self-perception, have long been known to be highly associated with depressive disorders (Jaycox et al., 1994; Bandura et al., 1999; Roberts, 1999; Roberts et al., 2008).

According to Seligman et al. (1984), children’s attribution styles often converge with that of their parents, especially their mothers. Similarly, an Australian study reported an association between parent attribution styles and children’s attributions concerning academic performance (Khodayarifard et al., 2010); whilst Lau et al. (2006) proposed that genetic and social factors combine to influence children’s attributions. Seligman explains that individuals with a negative attributional style attribute “bad” events to internal, stable, and global causes. For example, a child who performed poorly on a test may attribute their performance to their inadequate academic ability (internal), which they believe will always be the case (stable), and will be a contributing factor to poor performance on any other form of assessment (global). Such a negative attributional style can become so entrenched that an individual automatically interprets events within their attributional bias. This may present further problems as children transition to adolescence: a period believed crucial for the development of mental health problems; and a time when persistent patterns of lifestyle and behavior become established. This period corresponds with significant physical, biological, and social changes, and is associated with higher rates of depression (Stark et al., 2006; Reardon et al., 2009).

Socio-demographic, family mental health history, and normative developmental challenges may be beyond the scope of a psychological intervention, however, the cognitive sequelae associated with such factors may be the focus of treatment and preventive mental health interventions for children and adolescents. Although effective treatments are available, it is believed that many young people may not receive treatment due to the low rate of recognition and reporting of symptoms (Patterson et al., 2012). This is problematic for children at increased risk: with reports estimating that 30% of at risk children will develop clinical problems in adulthood compared with only 8% of low risk children (Durlak, 1995).

The importance of intervention with children and adolescents may be even greater in socially disadvantaged children, who are generally at higher risk for mental health problems (Zubrick et al., 1995; Hunter, 2007; Jackson and Goodman, 2011); and may have less access to health services (Zubrick et al., 1995). Durlak (1995) reported that primary intervention for socially disadvantaged children is a cost-effective way of reducing the incidence of internalizing disorders in adolescence; with universal interventions especially likely to help prevent a higher proportion of clinically diagnosable conditions. As such, implementing preventive approaches is considered preferable to delaying interventions until problems become recognized and reported. For sustainability and continuity to be maximized it has been argued that interventions based in the school are the most appropriate places for universal interventions to occur (Greenberg et al., 2001).

Preventive interventions for young people are often delivered in schools, which has the advantage of increasing the numbers of children receiving the treatment. Two commonly used approaches to preventive interventions include (i) Indicated, and (ii) Universal interventions. Indicated interventions specifically target children at risk for developing a disorder, whereas the universal interventions are delivered to entire cohorts of children regardless of current symptoms or risk status. Strong evidence exists for the preventive effect of indicated approaches: For example, Gillham and Reivich (1999) and Gillham et al. (1995) observed preventive effects for depression up to 2 years following intervention with the “Penn” program, and apparent cognitive effects continuing up to 3 years follow-up, when delivered to children at risk of depression.

Whilst indicated programs have demonstrated effectiveness in reducing anxiety and, to a lesser extent, depression symptoms, there are disadvantages associated with this approach. Indicated programs are often delivered by psychologists, increasing costs, and limiting accessibility in socially disadvantaged settings. In addition, children whose risk has not yet been identified do not receive the intervention (Barrett and Turner, 2001). Further, children who are removed from classroom to participate in indicated programs may experience stigma, which is known to be associated with anxiety and depressive symptoms (Roberts, 1999).

Universal programs have been recommended as they avoid such problems by providing a broad spectrum, non-targeted intervention, which enables all children to receive training in resilience skills without the social stigma of being identified or selected (Roberts et al., 2003; Huggins et al., 2008). Internationally, a universal program, called PATHS is the most comprehensive prevention program to date in the 6 years and upward age range that has had an influence on internalizing symptoms. Fostering emotional competency skills as well as social competency skills in PATHS program have been shown to have an impact on internalizing and externalizing programs, emotional understanding, emotional expression and emotion regulation, emotional competence and conduct problems when included in emotional and social competence programs for children as young as 5–6 years of age (Kam et al., 2004; Kelly et al., 2004). Support for the emphasis on social and emotional competence in younger child prevention programs has also been shown with the I can solve problems program (Shure and Spivack, 1982) and the incredible years classroom social skills and problem solving curriculum [Child Dinosaur Program (Webster-Stratton and Reid, 2004)]. However, PATHS and these other programs do not specifically target internalizing problems in children, and PATHS in particular is labor intensive, running weekly over a number of years.

In Australia, there have been several attempts to prevent anxiety and/or depression with children as young as 5 years old with mixed results. For example, Lock and Barrett (2003) investigated the effectiveness of FRIENDS program to prevent the development of anxiety and depressive symptoms in children aged from 6 to 16 years old. Overall findings indicated that in the longer-term the FRIENDS program was partly effective as there was significantly less high risk students in the intervention compared to the control group in the 36 month follow-up across all grades. However, children at risk for depressive disorders were not addressed, and effects for depressive symptomatology were rare. Comparing PATHS and FRIENDS, while FRIENDS program included a fear hierarchy and exposure feature that is targeted more at anxiety symptomatology and disorders (Kendall et al., 1992), PATHS did not include enactive programming and was not targeting internalizing disorders or symptoms. In support of this, Kendall et al. (1992) argued that to address co-morbid depressive and anxiety disorders from a cognitive-behavioral theoretical perspective, enactive programming should be included. This would incorporate the construction of a fear hierarchy and exposure to the feared stimulus for anxiety and for depression which would include the scheduling of pleasant events. The inclusion of enactive programming would maximize chances of addressing both types of internalizing disorders (Chu et al., 2012). A number of studies have shown that the behavioral exposure approach can be used to reduce anxiety symptoms and disorders in children as young as 4 years of age (Montenegro, 1968; Hirshfeld-Becker et al., 2010; Fox et al., 2012). For example Fox et al. (2012) reported that 3–5 year olds showing decreased child anxiety symptoms and improved emotional understanding skills following behavioral approach strategies to reduce their anxiety symptoms. In addition in Australia, Cobham (2012) showed that children as young as 7 years of age improved significantly on diagnostic interviews, anxiety questionnaires and behavioral checklists compared to children in the wait-list condition when behavioral approach strategies were used to reduce anxiety. These studies demonstrate that behavioral exposure techniques can aid in reducing anxiety and promote emotional resilience in children as young as 3 years of age. However, parents have often been involved in working with the younger children (3–7 years), and the trials have not generally been universal involving a large number of children.

The importance of emotional competence has been highlighted in recent research showing it is essential to the social, emotional and affective development of children and that it should be included in any programming targeting the prevention of anxiety and depression (Halberstadt et al., 2001; Buckley et al., 2003). Besides the PATHS program, there are more recent social and emotional programs for children as young as 4 years of age: The Head Start (Izard et al., 2008); The Roots of Empathy program (Schonert-Reichl et al., 2012). The “You Can Do it” Program (YCDI; Ashdown and Bernard, 2012). However, these programs have often not targeted internalizing problems and have not incorporated a range of enactive programing techniques such as pleasurable events scheduling, outcomes have not always included parent self-report, outcomes have been assessed only in the short-term (pre and post), and training only over a limited time period, for example 2 h in the case of YCDI. Two meta-analyses on social and emotional programs have demonstrated that the universal-school based approach have significant advantages for school children. A meta-analysis was conducted on 213 school-based universal social and emotional programs with 270,034 kindergarten to high school students (Durlak et al., 2011) and the second meta-analysis reviewed 180 studies on 277,977 kindergarten through year 8 students (Payton et al., 2008). Findings showed that these programs are more effective when the teachers and other school staff deliver the program at school as compared to external trainers (Durlak et al., 2011). In terms of effect sizes, it is likely that such programs will have improved effect sizes for internalizing problems (which have often been small) once the programs also target internalizing problems through the additional components which have been shown to reduce anxiety and depression such as enactive programming and exercise.

An Australian review examined the results of 42 randomized controlled trials of school-based universal interventions for depression with children aged 5–12 years, and adolescents aged 13–19 years (Calear and Christensen, 2010). Approximately half of the studies reported reduced levels of depressive symptoms at 12 month follow-up, however 33 of the studies reviewed were lacking in a control group, reducing the generalizability and validity of the findings. Further, the interventions described in the majority of the studies were delivered by mental health professionals and researchers. Ideally, universal programs delivered through schools should be delivered by teachers, as part of the school curriculum, to minimize costs, and increase the number of children receiving the intervention.

### THE AOP-PTS INTERVENTION

The Aussie Optimism Program-Positive Thinking Skills (AOP-PTS; Rooney et al., 2004) is a universal intervention designed to prevent depression and anxiety among children during the middle childhood years. This 10-module program is implemented in primary school years 4 and 5. It uses cognitive and behavioral intervention strategies and targets social, emotional, and cognitive risk and protective factors for anxiety and depression. The cognitive component teaches children to identify and challenge negative thoughts, such as those concerning the self, current life circumstances, and the future, that are known to contribute to depressive and anxiety symptoms (Beck et al., 1979; Kendall, 2007). In addition, children are taught to accurately identify, label and monitor their feelings (Stark, 1990). The social and behavioral component includes engagement in pleasurable events, practice with a fear hierarchy, as well as relaxation training (Stark, 1990; Kendall, 2007).

The AOP-PTS addresses the disadvantages of indicated programs targeting anxiety or depression symptoms. For example, the program is delivered to whole classrooms by regular classroom teachers who have received specific training in the interventions. Such an approach is more sustainable than employing psychologists; and classroom teachers can extend, generalize and reinforce the skills learned in the program throughout the children’s school day. This approach makes it particularly appropriate in areas of social disadvantage as it offers an intervention that may otherwise be inaccessible to this group. An additional advantage of the AOP-PTS is that it addresses both anxiety and depressive symptoms within the one program, which is of importance as evidence indicates that anxiety disorders frequently pre-date adolescent depressive symptoms and disorders (Roberts et al., 2008).

Although the AOP-PTS is a relatively new program, promising results have already been observed. In a small randomized controlled trial (pilot study), delivered by psychologists, Rooney et al. (2006) found the AOP-PTS was associated with significant reductions in depressive symptoms and more positive attributions at post-test, as well as fewer depressive disorders at 9 month follow-up. Further, a larger randomized control study (Rooney et al., 2013), involving 22 low SES primary schools in the Perth metropolitan region, supported the earlier findings. In this larger trial, the program was delivered by teachers to year 4 and 5 children. The study obtained self-reports and, where indicated, clinical diagnoses, of anxiety and depressive symptoms; as well as attributional styles, and compared with a matched control group of children receiving the regular health education curriculum. Compared to controls, the children receiving the AOP-PTS intervention reported reduced depressive symptoms following the intervention, although these results were not apparent at 6 and 18 month follow-up. Further, there were no observed effects of the intervention on anxiety symptoms or attributional style at 6 and 18 months following the intervention. The authors suggested the complexity of the cognitive component of this version of the AOP-PTS may have accounted for this finding. An alternative explanation may be that the children had yet to experience a sufficiently challenging situation that necessitated drawing upon the skills they had developed during the program. The transition from primary to high school may present an opportunity for the children to implement these skills.

The results provided early evidence that AOP-PTS is associated with resilience to depression in children from low SES backgrounds in the short-term, and may have the potential to prevent depressive disorders. The findings suggests that the AOP-PTS is helping children to think more optimistically, which in turn may be helping to reduce depressive symptomatology and clinical disorders in the short term. However, to adequately assess the stability of these results over time, and the impact on incidence of disorders, additional follow-ups are required, particularly those that cross important developmental transitions such as the move to secondary school. A recent follow-up study by Morrison et al. (2013) evaluated the medium term effectiveness of the AOP-PTS at a 30 month follow-up. Results indicated no significant differences between control or intervention groups regarding levels of anxiety, depression, and attributional styles. However, children in the intervention group were reported significantly less hyperactive behaviors compared to the children in the control group, as reported by their primary caregivers. These findings suggest that the AOP-PTS has the capacity to treat externalizing problems at a medium term effect. However, it is not until these long term effects are assessed, then the full prevention effects of the AOP-PTS can be determined.

### AIMS AND HYPOTHESES OF THE PRESENT STUDY

The present study aims to address this gap in the literature by analyzing data that has been collected as children transition to years 8 and/or 9 of high school: 42 and 54 months, respectively, following exposure to a universal preventive program. Self-report ratings of depression, anxiety, and attributions, completed by children who received the *AOP-PTS* in years 4 and 5 of primary school will be re-examined during years 8 and 9 and compared against ratings of children who received standard health education curriculum. If the AOP-PTS demonstrates efficacy effective in reducing risk for depression and anxiety, the following hypotheses may be drawn:

H1: Compared to the children in the control group, children who received the AOP-PTS program in year 4/5 will report lower levels of internalizing symptomatology (anxiety and depressive symptoms) at the 42 and 54 months follow-ups.
H1: Compared to the children in the control group, children who received the AOP-PTS program in year 4/5 will report more positive and less negative attribution style, and higher levels of optimism, [as measured by the children’s attributional style questionnaire (CASQ)] at the 42 and 54 months follow-ups.

## MATERIALS AND METHODS

### PARTICIPANTS

910 (89%) out of 1021 year 4 students from 22 primary schools received consent to participate in the pre-test assessment. The mean age of the students was 8.75 years (SD = 0.36); 51.4% (*n* = 467) were male and 48.6% (*n* = 442) were female. Due to attrition, the sample sizes for data collected at 42 and 54 months was 190 and 180 respectively. Child gender was not reported by 27 (14%) and 34 (19%) cases at 42 and 54 month follow-up, respectively; however, as earlier reports of this intervention (up to 18 month follow-up) found no significant gender by group by time interaction (Rooney et al., 2013), gender was not expected to impact on the 42 and 54 month follow-up results. Whilst the previous study also obtained clinical diagnoses for children at or beyond clinical cut-off on symptom measures, such information was not collected for the current study due to lower numbers.

### OUTCOME MEASURES

*The children’s depression inventory* (CDI; Kovacs, 1992) is a 27-item self-rated instrument for assessing symptoms of depression in children aged between 7 and 17 years. The CDI has been shown to have adequate psychometric properties across a range of populations. Kovacs reported that the measure has good internal consistency, with alpha reliability coefficients ranging from 0.71 to 0.89, and 1-week and 6-month test–retest reliability coefficients of 0.87 and 0.54 respectively. The CDI has good convergent validity with other self-report measures of depression (Cole and Turner, 1993). For this study, Item 9 (suicide ideation) was removed from the scale as school principals in the pilot study voiced concerns about the use of this item with children as young as 8 years. The total depression score in this study therefore ranged from 0 to 52, with higher scores indicating higher levels of depression symptoms. Rooney et al. (2006) reported a Cronbach’s alpha coefficient of 0.87 for the abbreviated CDI.

*Spence Children’s Anxiety Scale* (SCAS; Spence, 1998) is a 45-item self-report measure designed to assess anxiety symptoms in children. The SCAS was developed from community samples aged from 8 to 12 years; however, norms for clinical samples are also available. The measure has demonstrated high reliability, with Spence (1998) reporting an alpha coefficient of 0.92 and Guttman split-half coefficient of 0.90 in a community sample of over 2,000 8–12 year old children. The internal consistencies of the subscales range between 0.60 and 0.82 and the 6-week test–retest reliability of the total measure was found to be 0.60 when calculated with a smaller sample of 344 children in Spence’s (1998) study. The measure has also demonstrated concurrent validity as evidenced by high correlations with the Revised Children’s Manifest Anxiety Scale (RCMAS; Reynolds and Richmond, 1978).

*The CASQ* was developed by Seligman et al. (1984) for children 8 years and older. The measure has 48 self-rated items; 24 relating to positive events (CASQ-P) and 24 to negative events (CASQ-N). The alpha reliabilities range from 0.53 to 0.60 for the CASQ-P and from 0.45 to 0.46 for the CASQ-N (Thompson et al., 1998). Nolen-Hoeksema et al. (1986) reported test–retest reliabilities over periods of 3–12 months ranging from 0.61 to 0.35.

### INTERVENTION

*The AOP-PTS* (Rooney et al., 2004) was designed to meet the developmental needs of children in middle primary school, years 4 and 5. The intervention involved 10 weekly 60-min sessions based on the cognitive and behavioral principles outlined by Seligman et al. (1995). Teachers implementing the program, and all students receiving the intervention, received manuals and workbooks containing all necessary resources to successfully complete the program. The initial session focused on confidentiality and group rules. The remaining sessions have range of activities, roles plays, and games. The aspects of cognitive and behavioral skills focused on identifying thoughts and feelings, exploring the connection between thoughts, feelings and behaviors, evaluating and challenging thoughts, learning to think more accurately and positively, learning about relaxation and distraction, scheduling of pleasurable events, and constructing a fear hierarchy. Contents of the 10-modules are listed in **Table 1**. The program outcomes and activities were all designed to fit with the learning outcomes of the Western Australian Curriculum Framework Health Curriculum, and emphasized learning areas relating to interpersonal and self-management skills. The intervention was implemented by class-room teachers, with all children in the classroom participating.

**Table 1:** Content of positive thinking program.

Module	Title
Module 1	Introduction and planning for fun activities
Module 2	Identifying feelings and being brave
Module 3	Feelings, situations, and thoughts
Module 4	The thought feeling connection
Module 5	Helpful and unhelpful thinking
Module 6	Looking for evidence and thinking positively – brave hierarchy steps begin
Module 7	Think before you sink
Module 8	Challenging situations and thinking the worst
Module 9	Best, worst and most likely outcomes
Module 10	Being positive

### PROCEDURE

There were 12 randomly selected schools from the Canning and Swan districts and these schools are within the largest (top 50%) and poorest (bottom 30%) in the WA Department of Education and Training School Database. The 12 schools were matched to other schools in the data base in terms of SES, class size, and school size. Out of 24 matched schools, active and passive consents from 22 schools were received. The active – passive consent process resulted in 111 children refused to participate. The assessment was conducted by trained research assistants in a class with the presence of the class teachers. A standardized protocol was used in the assessment sessions and the children were informed that they can withdraw at any time without penalty. Their information will be keep confidential however if their response indicated any sign of distress, their parents will be informed to find further help. The research assistants read aloud the questionnaires in class and the assessment sessions took around 30–45 min to complete.

The 10-week class sessions was run by the class teachers who had received 8 h of training in a workshop run by the Aussie Optimism Team. The workshop covered on background information on children’s anxiety and depression. Teachers who completed the workshop, received a set of resources of teacher manual, student booklet, and parent booklet. The teachers’ manual incorporated the program rationale and link to the Curriculum Framework, content details, role plays, and activities. Teachers received supervision from the Aussie Optimism Team and the implementation’s integrity was recorded in the self-reported teachers’ log book. To further validate the implementation procedure, 25% of the 10-week sessions were observed by the trained research assistants. 88.46% of the teachers completed the checklist log book and the mean percentage of the contents covered were 97, 99, 98, 96, 98, 96, 94, 94, 92, and 92 (*M* = 95.60%, SD + 5.31%). Attendance rates indicated that each student completed an average of nine sessions (*M* = 9.03, SD = 2.143).

### RESEARCH DESIGN

Twelve schools were randomly selected from the largest and poorest schools in Western Australia districts of Swan and Canning. Each of the 12 schools was matched to another school from the same districts in terms of SES, class size, and school size. Out of the 24 matched schools, only 22 have agreed to participate. In each pair, one school was allocated to intervention condition and reciprocally for the other school. The year 4 students were assessed in four occasions:

## RESULTS

### STUDENT ATTRITION

Of the total of 910 children assessed at pre-test, observations were obtained from 190 and 180 at 42 and 54 month follow-up respectively. This represents an attrition rate of approximately 80 per cent of the pre- (*n* = 910) and post-intervention (*n* = 908) samples. Attrition rates did not differ between groups. Reasons for attrition were unavailable; although the relocation to new schools (from primary to high school) is likely a contributing factor. **Table 2** shows the number of participants in the intervention and control groups for each of the three assessment periods (pre-test, 42 month follow-up, and 54 month follow-up).

**Table 2:** Total number of participants in intervention and control groups at pre-test, 42-month, and 54-month follow-up.

	Intervention	Control
Pre-test	467	443
42-months	102	88
54-months	100	80

The means and standard deviations (SD) for CDI, SCAS, and CASQ scores obtained at pre-test and 42 and 54 month follow-up are presented in **Table 3**. The CASQ scores were grouped according to negative and positive attributions (CASQneg and CASQpos, respectively), and the total optimism score (CASQtot).

**Table 3:** Mean (SD) at pre-test, 42-month, and 54-month follow-up for intervention and control groups for each outcome measure.

	Intervention group		Control group	
	Mean	SD	Mean	SD
Pre-test CDI	11.99	9.692	11.71	8.370
42-months CDI	6.28	6.626	6.73	6.389
54-months CDI	6.98	7.182	5.86	5.763
				
Pre-test SCAS	32.62	20.985	30.19	17.465
42-months SCAS	32.01	10.311	30.89	10.165
54-months SCAS	29.85	11.178	30.41	10.454
				
Pre-test CASQneg	8.45	3.172	8.38	3.188
42-months CASQneg	14.26	2.400	13.20	2.809
54-months CASQneg	25.81	4.741	25.34	3.229
				
Pre-test CASQpos	13.90	3.012	13.75	2.983
42-months CASQpos	12.77	2.379	11.95	2.496
54-months CASQpos	13.53	2.890	13.21	2.260
				
Pre-test CASQtot	5.45	4.913	5.38	4.9499
42-months CASQtot	-1.49	2.954	-1.250	3.715
54-months CASQtot	-12.28	2.726	-12.12	1.925
				

### OUTCOME ANALYSIS

A multi-level mixed effects (MLM) linear regression model (Bryk and Raudenbush, 1987) was used to examine changes in the outcome measures. The regression model is “mixed” in the sense that it includes both random and fixed effects. There were two categorical random effects (student, school), one categorical fixed effect (group: intervention, control), and one ordinal fixed effect (time: pre-test, 42 month follow-up, 54 month follow-up). The regression model is “multi-level” in the sense that time is nested within student, and student is nested within school. The MLM regression model was implemented through SPSS’s generalized linear mixed models (GLMM: SPSS Version 19). In order to optimize the likelihood of convergence, a separate GLMM analysis was run for each of the outcomes (CDI, SCAS, CASQtot, CASQneg, and CASQpos).

There was no significant group by time interaction (*F*[2,1274] = 0.68, *p* = 0.507), and no main effect for group (*F*[1,1274] = 1.59, *p* = 0.208) on the measure of depression (CDI). The main effect for time, however, was significant (*F*[2,1274] = 52.63, *p* < 0.001). Least significant difference (LSD) *post hoc* contrasts conducted across the main effect for time indicated a significant reduction in depression between pre-test and 42 month follow-up (*p* < 0.001) although there was no subsequent change between 42 and 54 month follow-up (*p* = 0.378). The reduction observed between pre-test and 54-month follow-up was also significant (*p* < 0.001).

On the anxiety measure (SCAS) there was no significant group by time interaction (*F*[2,1274] = 1.78, *p* = 0.170), no main effect for group (*F*[1,1274] = 1.44, *p* = 0.231), and no main effect for time (*F*[2,1274] = 0.68, *p* = 0.509).

On the measure of optimism (CASQtot) there was no significant group by time interaction (*F*[2,1274] = 0.029, *p* = 0.865), and no main effect for group (*F*[1,1274] = 0.03, *p* = 0.865). There was a significant main effect for time (*F*[2,1274] = 1,141.89, *p* < 0.001). LSD *post hoc* contrasts conducted across the main effect for time indicated a significant decrease in optimism between pre-test and 42 month follow-up (*p* < 0.001) followed by a significant decrease between 42 and 54 month follow-up (*p* < 0.001). The reduction between pre-test and 54-month follow-up was, therefore, also significant (*p* < 0.001).

There was no significant group by time interaction (*F*[2,1274] = 1.81, *p* = 0.165), and no main effect for group (*F*[1,1274] = 3.02, *p* = 0.083) on the measure of negative attributions (CASQneg). The main effect for time, however, was significant (*F*[2,1274] = 1907.22, *p* < 0.001). LSD *post hoc* contrasts conducted across the main effect for time indicated a significant increase in negative attributions between pre-test to 42 month follow-up (*p* < 0.001) followed by a significant increase between 42 and 54 month follow-up (*p* < 0.001). The increase between pre-test and 54 month follow-up was, therefore, also significant (*p* < 0.001).

For the measure of positive attributions (CASQ pos) there was no significant group by time interaction (*F*[2,1274] = 0.89, *p* = 0.410), and no main effect for group (*F*[1,1274] = 2.85, *p* = 0.092). The main effect for time, however, was significant (*F*[2,1274] = 19.64, *p* < 0.001). LSD *post hoc* contrasts conducted across the main effect for time indicated a significant decrease in positive attributions from pre-test to 42 month follow-up (*p* < 0.001) followed by a significant increase from 42 to 54 month follow-up (*p* = 0.001); however, there was no significant change between pre-test and 54 month follow-up (*p* = 0.055).

## DISCUSSION

This aim of this study was to determine the long-term effectiveness of the AOP-PTS program in preventing anxiety and depressive symptoms, and increasing positive attributions, among 13–14 year old children in Perth, Western Australia. The 10-week AOP-PTS program was delivered to 8–9 year old children in years 4–5 of primary school, with follow-up assessment of internalizing symptoms and attributional styles conducted at 42 and 54 months post intervention. Data were also collected at 6 and 18 and 30 months post-intervention (Rooney et al., 2013; Morrison et al., 2013). Of these previous studies, the AOP-PTS program was found to be associated with reduced in depression symptoms at post-test, emotional difficulties at 6 months post-intervention but not at 18 months follow-up, and less hyperactive at 30 months follow-up (Morrison et al., 2013; Rooney et al., 2013).

For the current investigation, it was hypothesized that children who were exposed to the AOP-PTS program in years 4–5 of primary school would report lower levels of internalizing symptomatology, as indexed by measures of depression (CDI) and anxiety (SCAS), when compared to children in a matched control group following the transition to high school (years 8–9). Contrary to this hypothesis, there were no differences between the groups on either the CDI or SCAS at either 42 or 54 month follow-ups. A second hypothesis, that compared to matched controls children who had received the intervention would report more positive and less negative attributions (as measured by the CASQ), was also not supported. The final hypothesis, that children who had received the AOP-PTS program would demonstrate higher levels of optimism at 42 and 54 month follow-up compared with controls, was also not supported.

The lack of differences between groups may initially appear to suggest a failure of the AOP-PTS program to benefit this sample; however, changes evident on some of the outcome measures that were observed in both groups may help explain this. Firstly, children from both intervention and control groups reported fewer depression symptoms during their transition to high school (42 and 54 month follow-up) compared with their reports in years 4 and 5 of primary school. It appears that the level of symptoms had plateaued upon entering high school, however, as there were no further changes observed between the two follow-up periods. Other longitudinal studies have also observed a reduction in self-reported symptoms of depression (Nolen-Hoeksema et al., 1992; Twenge and Nolen-Hoeksema, 2002; LaGrange et al., 2008); and suggested this may be indicative of changes in cognitive development associated with the ability to introspect and abstract thinking styles.

Conversely, a decrease in self-reported optimism and a concomitant increase in self-reported negative attributional style were observed for all children at each of the follow-up periods. This is inconsistent with the theory proposed by Seligman et al. (1995) – that increased optimism and reduced negative attributions are associated with reduced risk of depression. Similarly, changes in self-reported positive attributions, which had decreased by the 42 month follow-up, but were restored by 54 month follow-up, are at odds with the reduced levels of depression. These results, however, are consistent with other reports in which expected correlations between self-reported depression symptoms and negative attributional styles in older children were not observed (Bennett and Bates, 1995; Abela and Sarin, 2002; LaGrange et al., 2008; Morris et al., 2008). Rather than reflecting a failure of the AOP-PTS program to reduce internalizing symptoms in children, it is possible that an explanation lies in theories associated with cognitive development and learning.

The cognitive component of the material may have been too complex for 8–9 year olds to fully assimilate. According to Piaget (1953) children of this age are considered to be in the “concrete operations” stage of cognitive development. Some of the concepts in the AOP-PTS may require the capacity for abstract reasoning, which is believed to develop from around age 11 years (Slee et al., 2012). The developers of the AOP-PTS have suggested simplification of the cognitive aspects for future versions of the program. The differences in capacity for processing abstract concepts may also have impacted upon children’s interpretations and responses to items in the assessment instruments. Whilst all the instruments were designed for use with children, and have demonstrated reliability and validity, they have not been evaluated for re-test reliability across intervals as long as 3 and 4 years. In addition to comparing follow-up responses to those obtained at pre-test, it may be useful to compare responses at each assessment occasion with age-related normative data. Furthermore, upon review the internal reliability of the CASQ is relatively low for most sub-scales and it is recommended that an alternative scale be developed to measure children’s attribution.

Although the cognitive aspect of the program may be too complex, children who received the intervention did demonstrate a benefit at post-intervention, 6 and 30 months follow-up (Rooney et al., 2013; Morrison et al., 2013), suggesting they were able to benefit from the program in the short and medium term. However, the single 10 week delivery of the program may have been insufficient to develop and consolidate learning to enable children to continue utilizing the skills for the longer term. According to Kolb (1984) there are four stages of learning of abstract concepts:

(1) Concrete Experience – during which a new experience of situation is encountered, or a reinterpretation of existing experience occurs.
(2) Reflective Observation (of the new experience). Of particular importance are reflections of any inconsistencies between experience and understanding.
(3) Abstract Conceptualization – during which reflection gives rise to a new idea, or a modification of an existing abstract concept.
(4) Active Experimentation – during which the learner applies the new ideas to the world around them to see what results.

Due to their concrete thinking styles, the 8–9 year old children involved in this study may have experienced difficulty engaging in stages two, three, and four beyond the initial delivery of the program, without specific structured opportunities to do so; resulting in reduced opportunities to consolidate the material. Further, Kolb and Kolb (2009) recommend that assessment of learned abstract concepts is best conducted with some delay following delivery of the material to be learned. The current format of the AOP-PTS program involved reviews at the end of each lesson. Whilst appropriate for the concrete stage of development, subsequent assessments beyond the period during which the program was delivered may have provided additional opportunities for children to progress through the stages of learning described by Kolb (1984), and, therefore, improve their ability to consolidate the material.

This is supported by Pavlik and Anderson (2005), who proposed that failure to continue to access the memories and/or use recently learned skills could result in skill degradation. Delivering the AOP-PTS program in its current 10 week format, and following up with regular reviews throughout the remainder of the primary school period, may have helped to ensure the skills conveyed in the AOP-PTS were better consolidated. Theories of skill acquisition not only recommend repeated practice when learning new skills, but suggest that in order to develop automaticity of a skill, the practice and rehearsal must continue for years (Ericsson et al., 1993). Indeed, Anderson (1982) proposed that 100 h was the minimum of time necessary to practice a new skill to develop proficiency.

Further, Shanteau (1992) suggested that the thinking styles associated with expertise may be domain specific. More specifically, Shanteau suggests that cognitive proceses are “tailored to the unique characteristics of a particular problem” (p. 13). In accordance with Shanteau, therefore, the children who received the AOP-PTS intervention would have benefited from opportunities to use the skills and concepts delivered in the classroom on multiple occasions and in multiple settings across the period from initial delivery to the point of follow-up 42 and 54 months later. With their more concrete type of cognitive processing, it may have been difficult for these younger children to abstract the classroom-delivered information to real-life situations outside the classroom.

For the purposes of this study, therefore, the immature cognitive development of 8–9 year olds, coupled with limited opportunities to practice and assess their skills repeatedly, and in novel situations, would support the suggestion that continued development and practice of the skills associated with the AOP-PTS program underlies the failure to observe the anticipated effects.

The AOP-PTS program guides young children to develop skills in observing, interpreting, and responding to their environment that may contrast with the habitual styles that they have learned through observation of significant others around them. For children whose environments model less optimism and more negative attributions, such as are believed to be associated with internalizing disorders, the use of a program such as AOP-PTS is likely to deliver an alternative way of viewing and responding to the world, and better equip children with the coping skills to minimize the risk of depression and anxiety disorders. However, based on theories of learning, development of expertise, and cognitive development, a single delivery of the program may not suffice for longer-term retention of the associated skill set.

## CONCLUSION

This study involved the investigation of the long-term effectiveness of the AOP-PTS program in reducing risk and incidence of internalizing disorders. For the purposes of the current study, the combination of complex abstract material, delivered at an age of concrete cognitive style, in the absence of repeated opportunities to consolidate the skills, in conjunction with the small sample size due to attrition, may underlie the failure to observe any benefits from this program at 42 and 54 month follow-up. This is not to suggest that the program does not have the potential for effectiveness in minimising risk of internalizing disorders: indeed benefits were reported immediately at post-intervention, 6 and 30 months follow-up (Morrison et al., 2013; Rooney et al., 2013), suggesting they were able to benefit from the program in the short and medium term. The results do suggest that future programs should consider the following: (i) modifying the complexity of the content to better suit the concrete cognitive style for younger children; (ii) revisit the content materials after 6-month lapse to revise the social and emotional skills learned. It is not necessary to do the entire 10-week program but recommended to engage in any selected activities and games on skills identified as needed; and (iii) provision of additional opportunities for children to consolidate their skills via additional presentations of age-appropriate material throughout primary and possibly early secondary school. The program teacher resource lists several other programs designed to suit children of different ages: an investigation ascertaining effectiveness after delivering each of these programs in sequence to the same group of children may provide a better indication of how well programs such as this can achieve the desired objectives.

## Conflict of Interest Statement

The authors declare that the research was conducted in the absence of any commercial or financial relationships that could be construed as a potential conflict of interest.
